# From Misdiagnosis to Recognition: Torsades de Pointes Masquerading as Non-sustained Ventricular Tachycardia

**DOI:** 10.7759/cureus.92354

**Published:** 2025-09-15

**Authors:** Sai Karthik Kommineni, John Sherret, Varshitha Kondapaneni, Venkata Vedantam, Vijay Ramu

**Affiliations:** 1 Cardiovascular Disease, East Tennessee State University Quillen College of Medicine, Johnson City, USA; 2 Internal Medicine, James H. Quillen Veterans Affairs Medical Center, Johnson City, USA; 3 Internal Medicine Residency, East Tennessee State University Quillen College of Medicine, Johnson City, USA; 4 Internal Medicine, East Tennessee State University Quillen College of Medicine, Johnson City, USA

**Keywords:** congenital long qt syndrome, ecg interpretation, kcne1 gene, non-sustained ventricular tachycardia, polymorphic ventricular tachycardia, qt prolongation, sudden cardiac death prevention, torsades de pointes, ventricular tachycardia

## Abstract

Torsades de Pointes (TdP) is a form of polymorphic ventricular tachycardia often mischaracterized as benign non-sustained ventricular tachycardia (NSVT). This arrhythmia is uniquely associated with a prolonged QT interval and can be precipitated by factors such as electrolyte disturbances (e.g., hypokalemia and hypomagnesemia) or medications that block cardiac potassium channels, as well as congenital long QT syndromes. TdP is considered one of the most dangerous cardiac arrhythmias due to its propensity to degenerate into ventricular fibrillation and cause sudden cardiac death. Prompt recognition and appropriate management are therefore critical. We present the case of a 57-year-old female whose recurrent polymorphic ventricular tachycardia was initially misidentified as NSVT, leading to potentially harmful management. With this case report, we aim to increase awareness of TdP and emphasize the importance of accurate electrocardiogram interpretation in preventing misdiagnosis and iatrogenic injury.

## Introduction

Ventricular tachycardia (VT) is defined as a tachyarrhythmia originating in the ventricles with a QRS duration >120 ms on ECG. VT is further classified by duration into non-sustained (<30 seconds) or sustained (≥30 seconds), and by morphology into monomorphic or polymorphic VT [1]. Torsades de Pointes (TdP) is a specific type of polymorphic VT characterized on ECG by a gradual change in QRS amplitude and a twisting of the QRS complexes around the isoelectric baseline [2]. Importantly, TdP occurs in the setting of a prolonged QT interval (QTc >450 ms in males or >460 ms in females) [2]. QT prolongation can be congenital or acquired. The two classic congenital syndromes are Romano-Ward (autosomal dominant) and Jervell and Lange-Nielsen (autosomal recessive with congenital deafness). Although the true prevalence of congenital long QT syndrome is not fully known, estimates range from ~1 in 2,000 to 1 in 20,000 persons [3]. Acquired causes of QT prolongation are more common and are usually drug-related. A wide variety of medications are associated with prolonged QT and TdP, most notably antiarrhythmics, antipsychotics, antiemetics, antifungals, and certain antimicrobials. Common examples include amiodarone (antiarrhythmic), haloperidol (antipsychotic), ondansetron (antiemetic), fluconazole (antifungal), and macrolide antibiotics such as azithromycin [3,4]. Additional risk factors for developing TdP include female sex, age >65 years, bradycardia, structural heart disease, and electrolyte disturbances (especially hypokalemia, hypomagnesemia, and hypocalcemia) [3]. Given the potential lethality of TdP, clinicians must maintain a high index of suspicion for this arrhythmia when evaluating patients with polymorphic VT or prolonged QT, so that appropriate therapy can be instituted without delay. This case report aims to illustrate a rare clinical scenario in which TdP was initially misdiagnosed as non-sustained VT, emphasizing the importance of accurate ECG interpretation, recognition of QT prolongation, and timely corrective interventions to prevent malignant arrhythmias and sudden cardiac death.

## Case presentation

**Figure 1:**
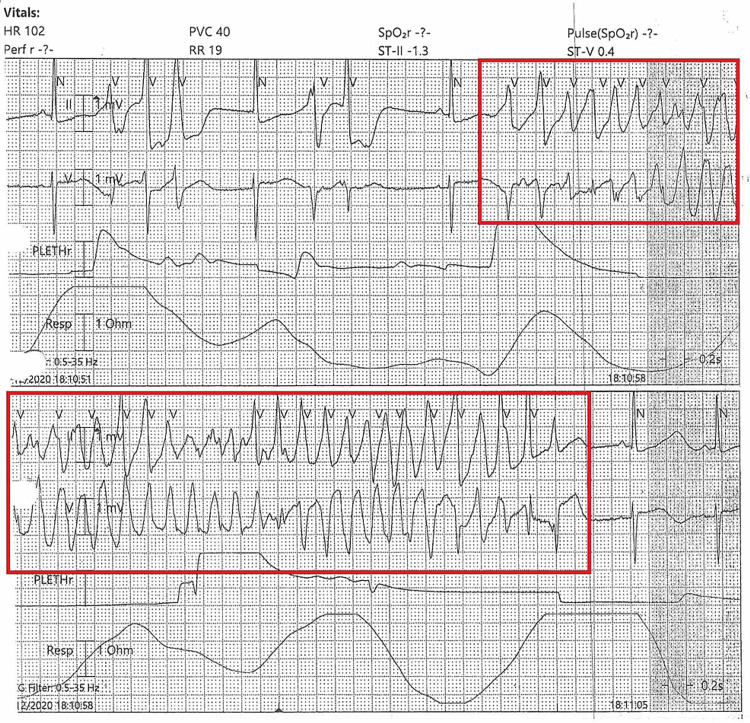
Initial ECG at the outside hospital emergency department, showing a polymorphic ventricular tachycardia with wide QRS complexes (torsades de pointes appearance). The red box highlights torsades de pointes, a polymorphic ventricular tachycardia, which was misinterpreted as a non-sustained ventricular tachycardia.

A 57-year-old female with a history of hypertension, hyperlipidemia, Crohn’s disease, tobacco use, and a prior deep vein thrombosis (on chronic apixaban) was transferred to our facility for evaluation of intermittent VT. One week before, she had undergone an exploratory laparotomy for Crohn’s disease, complicated by an incisional wound abscess that required incision and drainage. In the interim, she experienced episodes of palpitations and was found at an outside hospital to be in a VT rhythm. The arrhythmia was described as intermittent over several weeks with increasing frequency. On presentation to our emergency department, the telemetry rhythm was interpreted as runs of non-sustained VT. The initial ECG (from the outside hospital) is shown in Figure 1, which demonstrates a polymorphic VT with wide QRS complexes.

**Figure 2:**
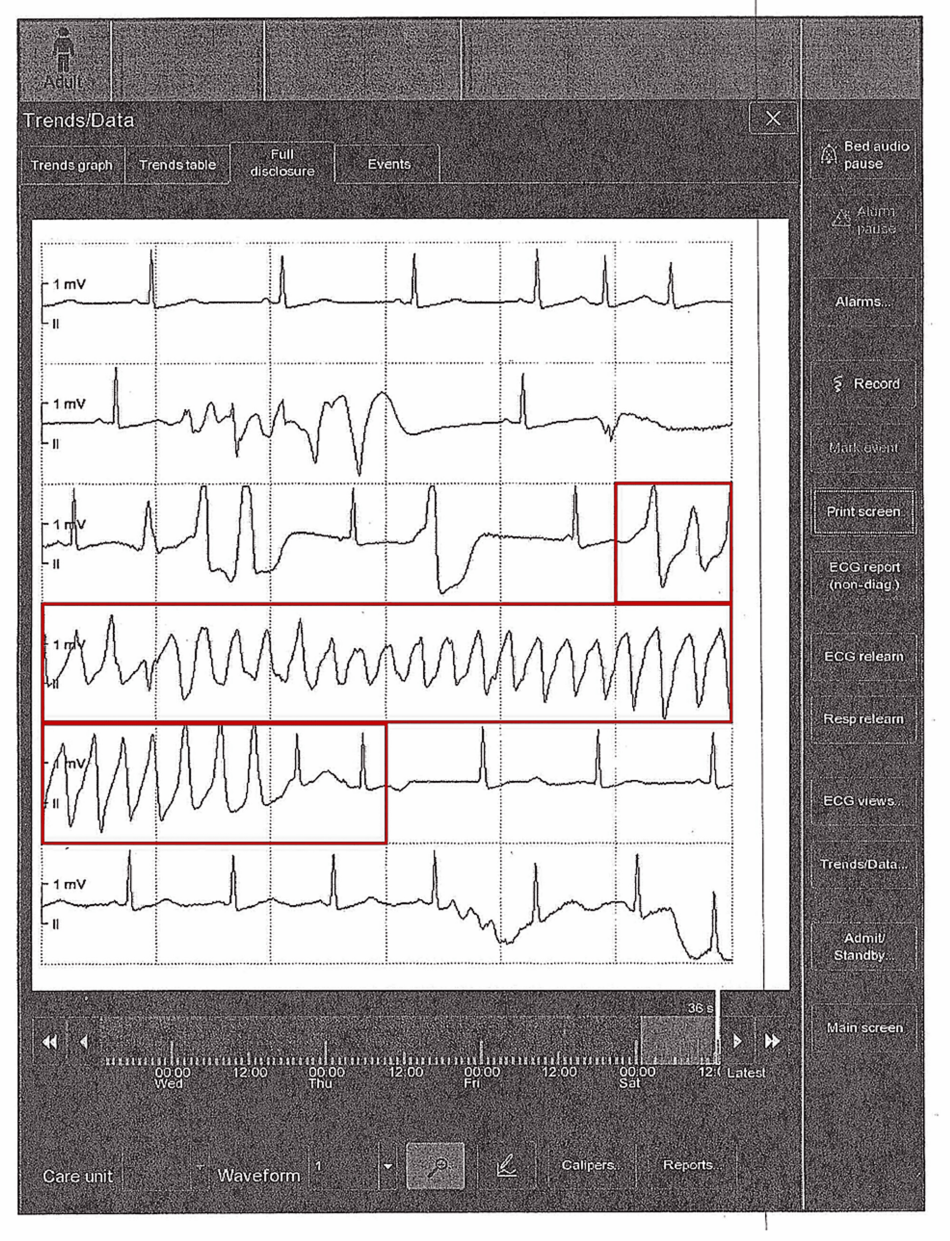
Telemetry strip showing torsades de pointes, a polymorphic VT with oscillating QRS amplitude, creating the classic “twisting of the points” morphology. The red box highlights torsades de pointes, a polymorphic ventricular tachycardia.

In the emergency department, the patient was hemodynamically stable. She was given an IV bolus of amiodarone 150 mg for presumed VT and started on a continuous amiodarone infusion. Laboratory results on arrival revealed a potassium of 4.2 mEq/L, magnesium of 1.7 mEq/L (low), and calcium of 9.8 mg/dL. On cardiac monitor, the underlying rhythm was sinus with frequent premature ventricular contractions (PVCs) and recurrent runs of polymorphic NSVT. Upon review of telemetry strips, the “NSVT” episodes were recognized to be TdP manifesting as polymorphic VT with an oscillating QRS axis (Figure 2).

During our initial evaluation on arrival, the patient was resting comfortably and denied any active symptoms. Her vital signs were notable for a heart rate of 80-90 bpm and blood pressure of 110/70 mmHg. She reported no chest pain, dyspnea, dizziness, or syncope at that time. She had no known personal cardiac history and no family history of sudden cardiac death or inherited arrhythmias. Given the recognition of torsades, we promptly administered 2 g of IV magnesium sulfate. Following these interventions, the ventricular ectopy frequency gradually decreased and the runs of VT subsided; however, the patient developed sinus bradycardia at 40-50 bpm. A follow-up 12-lead ECG now showed significant QT prolongation (QTc >480 ms). At this point, the continuous amiodarone infusion was discontinued due to concern that it could further prolong the QT interval and exacerbate the arrhythmia. The patient was transferred to the intensive care unit (ICU) for close monitoring.

In the ICU, despite correction of magnesium, the patient continued to have frequent PVCs and recurrent runs of polymorphic VT, with some non-sustained episodes lasting up to 20-28 beats. An infusion of IV lidocaine was initiated for antiarrhythmic management. The initial lidocaine rate produced little change after ~30 minutes, so the infusion rate was increased. Shortly thereafter, the PVCs completely resolved, and the VT episodes ceased. Given the history of unexplained torsades in a relatively young patient with minimal obvious triggers, we pursued an evaluation for congenital long QT syndrome. A genetic testing panel was sent, and the patient was started on oral mexiletine as a preventive antiarrhythmic measure. Throughout hospitalization, she experienced no further torsades episodes after the lidocaine was optimized. The option of placing an implantable cardioverter defibrillator (ICD) for secondary prevention was discussed; however, due to her recent abdominal wound infection, ICD implantation was deferred. Instead, a wearable defibrillator (LifeVest®) was provided at discharge, with a plan to reassess for permanent ICD placement once cleared by infectious disease team.

**Figure 3:**
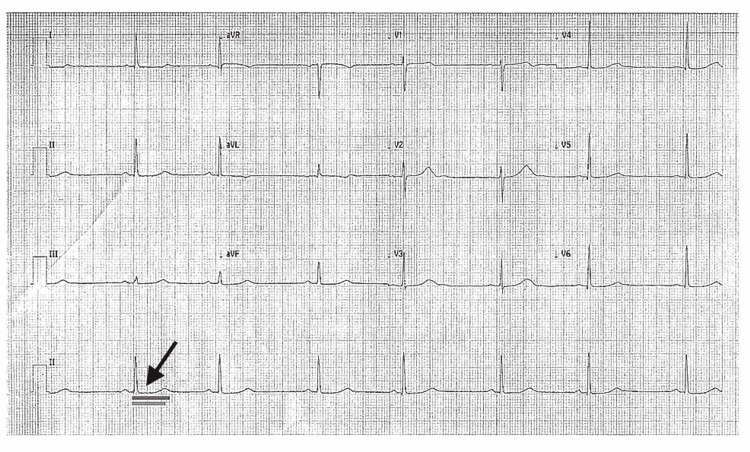
ECG on the day of discharge, demonstrating sinus bradycardia with a prolonged QTc of 481 ms (dark gray bar indicates the measured QT interval from the start of the Q wave to the end of the T wave; light gray bar indicates the upper limit of normal QT interval of 460 ms for females). The arrow corresponds to the prolonged QT interval, which was measured before discharge.

By the time of discharge, the patient’s ECG remained notable for a prolonged QT interval (Figure 3). Figure 3 illustrates a representative lead showing a measured QT interval of 533 ms, with a rate-corrected QTc of 481 ms (exceeding the normal female upper limit of 460 ms). This confirmed persistent long QT.

**Figure 4:**
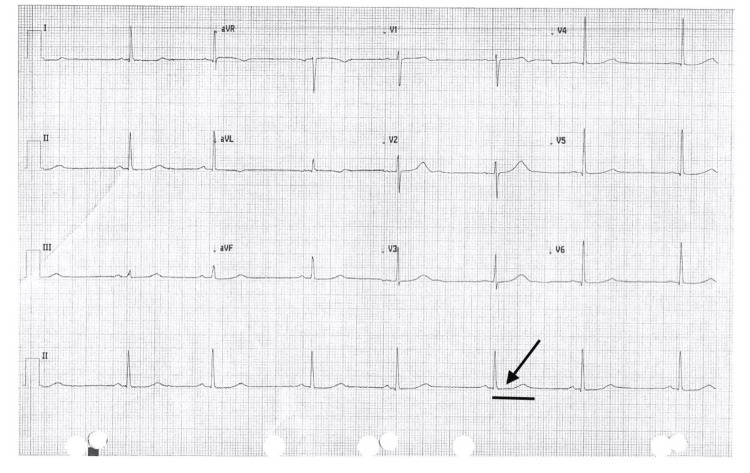
The most recent follow-up ECG demonstrates a prolonged QT interval (>480 ms), as marked by the black solid bar, suggestive of persistent QT prolongation. The arrow corresponds to a prolonged QT interval on the most recent clinic follow-up visit.

The patient continues to be observed in routine annual follow-up visits in the clinic, and Figure 4 illustrates a persistently prolonged QT interval (>480 ms) on the most recent ECG.

## Discussion

This case illustrates the importance of recognizing TdP, which requires clinical acumen, knowledge of precipitating factors, and careful ECG interpretation. Clinically, TdP can be variable in presentation. Many patients may be asymptomatic during runs of torsades, while others experience palpitations, lightheadedness, syncope, or even hemodynamic collapse [5]. In some reports, up to 50% of torsades cases are asymptomatic, and in about 10% of patients, the first presentation is sudden cardiac arrest. Our patient, notably, had very minimal symptoms despite frequent NSVT episodes. This highlights that the absence of symptoms does not rule out a malignant arrhythmia.

Medication history and risk factor assessment are critical when torsades is suspected. Multiple drug classes are known to induce QT prolongation and TdP, including antiarrhythmic drugs (Class IA such as procainamide; Class III such as sotalol and dofetilide), antipsychotics (e.g., haloperidol and quetiapine), certain antidepressants, antiemetics (e.g., ondansetron), antifungals (e.g., fluconazole and ketoconazole), and various antibiotics (especially macrolides and fluoroquinolones) [4]. Any newly added or high-dose medication with QT-prolonging potential should raise concern for TdP in a patient with a compatible arrhythmia. In our case, the patient was given amiodarone (a Class III antiarrhythmic) before TdP was recognized. Amiodarone can prolong repolarization and is generally not recommended in TdP, as it may aggravate QT prolongation. Fortunately, our patient did not deteriorate, but this underscores the risk of treating the “wrong” rhythm due to misdiagnosis. In addition to drugs, clinicians should evaluate for other precipitating factors such as hypokalemia, hypomagnesemia, bradyarrhythmias, or acute ischemia, all of which can contribute to TdP. Our patient had hypomagnesemia on presentation, which likely facilitated the development of torsades in the setting of her genetic predisposition.

On ECG, TdP is distinguished by its polymorphic QRS morphology and characteristic twisting axis. Unlike monomorphic VT, which has uniform QRS complexes, TdP shows QRS complexes of changing amplitude and polarity that appear to “twist” around the baseline. The ventricular rate during TdP is typically 200-300 beats per minute. The QRS complexes are wide (≥0.12 s) due to their ventricular origin, and atrioventricular dissociation is usually present (P waves, if visible, bear no fixed relation to the QRS complexes) [6]. TdP is often triggered by an early afterdepolarization, which is an abnormal electrical impulse that occurs before the heart has fully reset from the previous beat. On an ECG, this appears as a PVC falling on the T wave of the preceding beat, a finding known as the “R-on-T” phenomenon [7]. In our patient’s telemetry strips, we observed the telltale pattern of a short-long-short sequence preceding the onset of polymorphic VT, consistent with an R-on-T trigger. Another important ECG clue is the presence of a prolonged QT interval in the baseline rhythm, which helps differentiate torsades from other forms of polymorphic VT (such as those due to acute ischemia, which usually have normal QT). In this case, once the patient’s rhythm slowed, her QTc was prolonged to 481 ms (Figure 3), confirming the diagnosis of TdP in the context of long QT syndrome.

The mainstay of acute treatment for TdP involves eliminating any precipitating causes and stabilizing the cardiac membrane. First-line therapy is IV magnesium sulfate, regardless of the patient’s serum magnesium level. Magnesium suppresses early afterdepolarizations and is highly effective at terminating TdP or preventing its recurrence. A typical regimen is 2 g IV magnesium given as a slow bolus, followed by an infusion if needed. Concurrently, any QT-prolonging medications should be discontinued, and electrolyte imbalances must be corrected. If the patient is hemodynamically unstable (e.g., hypotensive, or in polymorphic VT lasting longer and not self-terminating), immediate synchronized cardioversion is indicated. In a pulseless scenario (TdP degenerating into ventricular fibrillation), defibrillation should be performed promptly [3,5].

For patients who remain in torsades or have frequent recurrent episodes despite magnesium and correction of risk factors, increasing the heart rate can be an effective strategy. Accelerating the heart rate shortens the QT interval and can prevent the pause-dependent initiation of TdP. This can be achieved with beta-agonist therapy such as isoproterenol infusion (titrated to a heart rate of 90-100 bpm) or via temporary overdrive pacing. It is important to note, however, that isoproterenol is contraindicated in congenital long QT syndrome, as it can paradoxically worsen QT prolongation in that setting. Overdrive transvenous pacing is therefore the preferred option if bradycardia-induced TdP is a concern in congenital cases [3]. In our patient, episodes of torsades occurred in the context of bradycardia (after amiodarone, her sinus rate dropped significantly). Although we did not need to initiate transvenous pacing, the use of lidocaine and maintenance of a slightly higher heart rate (by avoiding excessive beta-blockade) likely helped suppress further episodes. Lidocaine, a Class IB antiarrhythmic, can be useful in acquired TdP, as it shortens action potential duration and can terminate polymorphic VT in some cases [8]. Indeed, our patient’s arrhythmia responded to an increased dose of lidocaine after magnesium alone was insufficient. This aligns with prior reports that lidocaine may help suppress TdP, particularly when ischemia or myocardial scarring is involved in the arrhythmia’s etiology [8]. Importantly, any patient with TdP should be closely monitored in an ICU or cardiac care setting until the arrhythmia is fully resolved and the QT interval has normalized or the patient is adequately protected (e.g., by pacing or an implanted defibrillator) [8].

In the case described, the initial misdiagnosis of torsades as “monomorphic NSVT” led to a delay in appropriate therapy and the use of amiodarone, which could have worsened the situation. This underscores a key lesson: visual inspection of the rhythm strip and ECG by a knowledgeable clinician is crucial whenever an abnormal rhythm is reported. One should not rely solely on over-the-phone telemetry interpretations or automated ECG readings, especially for complex arrhythmias. Polymorphic VT should always prompt measurement of the QT interval to distinguish TdP from other causes. In our scenario, once torsades was recognized, the team swiftly changed management, resulting in a good outcome. Earlier recognition, however, would have avoided exposing the patient to amiodarone and might have prevented some of the recurrent episodes. This case also highlights the value of an interprofessional approach; the vigilance of nursing staff and telemetry technicians in flagging unusual ECG tracings, combined with pharmacists reviewing medication profiles, can contribute to catching a diagnosis of TdP before it is missed.

Genetic testing in our patient ultimately revealed a likely pathogenic variant in the KCNE1 gene. KCNE1 encodes a regulatory β-subunit of the cardiac potassium channel responsible for the slow delayed rectifier current (IKs). Loss-of-function mutations in KCNE1 impede the flow of potassium out of cardiomyocytes during repolarization, thereby prolonging the action potential and the QT interval. Heterozygous KCNE1 mutations cause an autosomal dominant form of long QT syndrome (often referred to as LQT5), which accounts for only 1%-2% of congenital long QT syndrome cases [9]. When KCNE1 mutations occur in both alleles (biallelic), they can lead to the autosomal recessive Jervell and Lange-Nielsen syndrome, which is characterized by profound congenital deafness in addition to long QT [10]. In our patient, three additional variants of uncertain significance were identified in the SCN5A, JUP, and TTN genes, but their clinical significance is unclear. The identification of a pathogenic KCNE1 variant, however, provides a unifying explanation for the patient’s TdP in the context of congenital long QT syndrome. This genetic confirmation has important implications: patients with congenital long QT syndrome often require long-term management with beta-blockers and an ICD for prevention of sudden death, and family screening is recommended. Our patient was discharged with a wearable defibrillator and plans for definitive ICD placement, as well as genetic counseling for her family.

## Conclusions

Non-sustained VT is a common occurrence in hospitalized patients, but not all VT is benign. This case demonstrates that TdP, a life-threatening polymorphic VT, can masquerade as brief runs of VT and may be initially overlooked, especially if the patient is asymptomatic. Under recognition or misdiagnosis of TdP can result in inappropriate treatment and expose the patient to a high risk of sudden cardiac death. To maximize patient safety, clinicians must maintain a high suspicion for torsades in any polymorphic VT, particularly in patients with known risk factors or QT-prolonging medications. Comprehensive care and communication among the healthcare team (nurses, telemetry technicians, pharmacists, and physicians) are vital to promptly identifying the distinctive ECG features of TdP and ensuring immediate management. In practice, this means visually confirming telemetry alarms and QT intervals on the ECG rather than relying solely on automated or reported interpretations. By doing so, one can avoid missing this dangerous arrhythmia or causing iatrogenic harm. Early recognition and appropriate therapy for TdP can be truly life-saving, as timely magnesium administration, removal of offending agents, and protective measures (such as pacing or defibrillation) will effectively prevent degeneration into ventricular fibrillation and cardiac arrest.
